# Re-Analysis of Abdominal Gland Volatilome Secretions of the African Weaver Ant, *Oecophylla longinoda* (Hymenoptera: Formicidae)

**DOI:** 10.3390/molecules26040871

**Published:** 2021-02-07

**Authors:** Bethelihem Mekonnen, Xavier Cheseto, Christian Pirk, Abdullahi Yusuf, Sunday Ekesi, Emilie Deletre, Baldwyn Torto

**Affiliations:** 1 International Centre of Insect Physiology and Ecology (*icipe*), Nairobi 30772-00100, Kenya; bmekonnen@icipe.org (B.M.); xcheseto@icipe.org (X.C.); sekesi@icipe.org (S.E.); 2 Department of Zoology and Entomology, University of Pretoria, Private Bag X20, Hatfield, Pretoria 0028, South Africa; cwwpirk@zoology.up.ac.za (C.P.); aayusuf@zoology.up.ac.za (A.Y.); 3 CIRAD, UPR HortSys (Agroecological Functioning and Performances of Horticultural Systems), University of Montpellier, 34000 Montpellier, France

**Keywords:** weaver ant, biological control, hydrocarbons, solid-phase microextraction (SPME), mass spectrometry

## Abstract

The African weaver ant, *Oecophylla longinoda*, is used as a biological control agent for the management of pests. The ant has several exocrine glands in the abdomen, including Dufour’s, poison, rectal, and sternal glands, which are associated with pheromone secretions for intra-specific communication. Previous studies have analyzed the gland secretions of Dufour’s and poison glands. The chemistry of the rectal and sternal glands is unknown. We re-analyzed the secretions from Dufour’s and poison glands plus the rectal and sternal glands to compare their chemistries and identify additional components. We used the solid-phase microextraction (SPME) technique to collect gland headspace volatiles and solvent extraction for the secretions. Coupled gas chromatography–mass spectrometry (GC-MS) analysis detected a total of 78 components, of which 62 were being reported for the first time. These additional components included 32 hydrocarbons, 12 carboxylic acids, 5 aldehydes, 3 alcohols, 2 ketones, 4 terpenes, 3 sterols, and 1 benzenoid. The chemistry of Dufour’s and poison glands showed a strong overlap and was distinct from that of the rectal and sternal glands. The different gland mixtures may contribute to the different physiological and behavioral functions in this ant species.

## 1. Introduction

Chemical communication is a well-documented phenomenon in ants [1], used within and outside the nest [2] for recruitment [3], defense [4], alarm [5], nestmate [6], and sexual recognition [7]. Ant semiochemicals are primarily released from different glands, for example, the Pavan gland located in the abdomen of ants in the families Dolichoderinae and Aneureinae [8], Dufour’s gland in harvester ants, [3], the sternal gland in African stink ants (*Pachycondyla tarsata*) [9], and the hindgut of the jet ant (*Lasius fuliginosus*) [10].

Weaver ants of the genus *Oecophylla* consist of two extant species, *O. longinoda* and *O. smaragdina*, distributed in the old-world tropics and known for sophisticated nest-building behaviors [11]. These ants possess different glands located in the abdomen, which are used for various purposes [12,13]. The poison gland of *Oecophylla longinoda* contains a large quantity of formic acid, whereas in *O. smaragdina*, the key component in the gland secretion is undecane [13,14]. Dufour’s gland, in both species, contains a mixture of hydrocarbons, including undecane and other n-alkanes (decane, dodecane, pentadecane, heptadecane, nonadecane, heneicosane, docosane, and tricosane) [13,14]. The two major compounds in the glands, formic acid and undecane, are used as an alarm/defense system in *O. longinoda* [13]. The functions of the gland secretions have not been well studied in *O. smaragdina.* Two more glands are reported in *O. longinoda*: the rectal gland releases pheromones that mediate recruitment to new food sources [12], whereas emigration to new sites and short-range recruitment to territorial intruders are facilitated by secretions from the sternal gland [12].

Previous studies have identified 23 compounds in Dufour’s gland secretions, with two compounds identified in the poison gland [13]. This study, which was carried out over four decades ago, used less sensitive analytical chemical techniques based on mass spectrometry fitted with packed glass columns and degradative reactions of isolates obtained from preparative chromatographic analysis to identify compounds. A recent study using modern and more sensitive analytical chemical techniques provided a comprehensive comparison of the chemical profiles of the cuticle, Dufour’s glands, poison glands, the head, headspace volatiles, and trails of a related species, *O. smaragdina* [14]. In this study, a total of 59 compounds were identified from these glands of worker ants, including aldehydes, alcohols, carboxylic acid, esters, fatty acids, terpenes, and hydrocarbons. Given these findings for *O. smaragdina*, we hypothesized that additional potential semiochemicals may be released in the abdominal gland secretions of *O. longinoda*.

Thus, in the present study, we re-analyzed the secretions of Dufour’s and poison glands to identify additional components. Further, since little is known about the composition of the sternal and rectal glands, we explored the chemistry of their secretions.

## 2. Results

Our results demonstrate that the use of more than one technique to collect gland secretions, followed by gas chromatography–mass spectrometry (GC-MS) analysis, reveals a complex blend of compounds, including chemical signatures associated with the different glands. Interestingly, GC-MS analysis detected a total of 78 components from secretions of the four glands, of which 62 are reported for the first time (presented in bold font in Table 1 and Table 2). Of these 62 additional components, the identities of 28 were confirmed with authentic samples. Quantitative variations in the detected components are shown in Table 1 and Table 2. These additional components include a complex mixture of 32 hydrocarbons (51.6%), dominated by unsaturated alkanes, which ranged in chain length from 13 to 25 carbon atoms; saturated alkanes of chain length from 6 to 31 carbon atoms were also detected, as were 12 carboxylic acids (19.4%), dominated by short-chain fatty acids of chain length from two to seven carbon atoms; 5 aldehydes (8.1%); 3 alcohols (4.8%); 2 ketones (3.2%); 4 terpenes (6.5%); 3 sterols (4.8%) and 1 benzenoid (1.6%). Solvent-extracted glands and headspace volatiles gave similar profiles, dominated by hydrocarbons, which were also identified in the gland secretions of *O. smaragdina* [14]. However, we found that the headspace volatiles were richer in fatty acids than the solvent-extracted glands. These data suggest further investigation into the potential pheromonal roles of these fatty acids and possibly the terpene *p*-cymene [13] and caryophyllene detected in the headspace volatiles. The patterns of the composition of the hydrocarbons were similar for both Dufour’s and poison glands. Likewise, the compositions of the rectal and sternal glands were similar, comprising saturated alkanes of chain length from 8 to 31 carbon atoms.

We found that undecane, heneicosane, and tricosane in Dufour’s gland secretions formed ~70% of the composition. Our results agree with previous work that reported these three compounds as the major components of the secretions of Dufour’s gland of *O. longinoda* [13], which act as alarm pheromones for the ant. It is possible that the low vapor pressures associated with some of these hydrocarbons, especially those ranging in chain length between 25 and 31 carbon atoms could help moderate the volatility of other components in Dufour’s gland secretions. Comparing our results to those reported by Bradshaw et al. [13], we identified seven classes of chemicals (hydrocarbons, carboxylic acids, terpenes, aldehydes, alcohols, ketones, and benzenoid), whereas these authors identified only two classes of chemicals (hydrocarbons and ester), comprising 23 compounds in Dufour’s gland of *O. longinoda*. This confirms the efficiency of our method (combining solid-phase microextraction (SPME) and solvent extraction) for both qualitative and quantitative analysis of gland secretions compared to the use of solvent extraction alone. Of the 23 compounds identified in [13], 22 were hydrocarbons (decane, undecane, dodecene, dodecane, 4-tridecene, tridecane, tetradecane, pentadecene, pentadecane, hexadecane, 8-heptadecene, heptadecane, octadecane, nonadecane, eicosane, heneicosene, heneicosane, docosane, tricosene, tricosane, pentacosane, and 4,7-heptadecadiene). The ester was partially identified as a hexadecyl ester. Notably, we did not identify any ester in Dufour’s gland secretions, although we found several carboxylic acids and alcohols detected in low quantities. As such, it is possible that given the right enzymes, the population of *O. longinoda* used in the current study or another population can make esters in Dufour’s gland secretions. Such differences in chemical signatures between populations may also be influenced by diet, geographic location, season, and environmental factors. Our results are in agreement with those of [13] and those found for the related species, the Asian weaver ant, *O. smaragdina* [14,15], which showed that the composition of Dufour’s gland secretions is dominated by the hydrocarbons undecane, heneicosane, and pentadecane, constituting more than 60% of the gland components (Table 1). Thus, it appears that the presence of undecane, heneicosane, and pentadecane in a higher proportion in Dufour’s gland secretions may serve as a generic chemical signature for these ant species, with the other constituents (ratio and concentration) providing the chemical profile for inter- and intra-species distinction, though further research is necessary to confirm this.

Interestingly, carboxylic acids have previously been detected in Dufour’s gland of workers of the crazy ant, *Paratrechina longicornis* [16]. The role of these carboxylic acids in this ant species is unknown, although to the best of our knowledge, this is the first study to identify carboxylic acids in Dufour’s gland of *O. longinoda.* As previously observed, the gland secretions of certain ant species also contain aldehydes and terpenes, for instance, the black garden ant, *Lasius niger* [17]; the black-headed bull ant, *Myrmecia nigriceps*; the giant bull ant, *M. gulosa* [18]; and the Sahara Desert ant, *Cataglyphis bicolor* [19]. Likewise, alcohols and ketones have been reported in the blood-red ant, *Formica sanguinea* [20]. Further studies are required to determine the role of these classes of compounds in the biology of ants. The specific chemical signature of the poison gland secretion dominated by the hydrocarbons undecane, tricosane, and heptacosane and the carboxylic acid formic acid (Table 1 and Table 2), identified in the present study, seems to agree with the composition previously reported [13]. Formic acid is one of the components of the alarm pheromone in *O. longinoda* [13]. However, Bradshaw et al. [13] partially identified a compound described as a derivative of 1-hexadecanol in the poison gland. On the other hand, in the present study, we identified the carboxylic acid derivative hexadecanoic acid of this alcohol in the Dufour’s gland but not in the poison gland secretion. This suggests that further studies to investigate the composition of *O. longinoda* gland secretions from different populations are necessary to enhance our understanding of gland chemistry. Nonetheless, our results agree with the composition of hydrocarbons recently reported for the poison gland secretions of the Asian weaver ant, *O. smaragdina*, which identified undecane, tricosane, and heneicosane as the most abundant components of the gland secretions [14], and formic acid previously identified as a major component of the poison gland secretions of carpenter ants (*Camponotus* spp.) [21]; the lemon ant, *Myrmelachista schumanni* [22]; and the red wood ant, *Formica rufa* [23].

Chemical analysis of the rectal gland of *O. longinoda* located in the hindgut revealed a range of hydrocarbons (Table 1). The major compounds identified were tricosane, undecane, and heneicosane. The gland has no specialized glandular tissue but is comprised of glandular cells responsible for pheromone production [4]. However, in the present study, since the entire hindgut was examined to access the glandular cells, there was a possibility of extracting products associated with cuticular components and digested food. Nonetheless, similar straight-chain hydrocarbons had previously been reported from the anal gland secretions of the ant *Novomessor cockerelli* [24].

Likewise, the sternal gland of this ant species does not have a reservoir; instead, the gland is composed of a group of glandular cells whose ducts penetrate the sclerotized cuticle [13]. The compounds identified in the sternal gland secretions included mainly hydrocarbons (Table 1), in agreement with previous results reported for the sternal gland secretions from the European paper wasp, *Polistes dominulus* [25]. The sternal-specific components heptane, α-cedrene, and, especially, dimethyl-branched saturated components, which were not detected in the other gland secretions, could be associated with cuticular components. Such differences need further investigation. Consequently, because of the approach we used to obtain sternal and rectal gland secretions, and to rule out the possibility of the presence of additional artifacts in our analysis, we did not carry out headspace collections and analysis of secretions of these glands.

## 3. Materials and Methods

### 3.1. Chemicals and Standards

Synthetic standards of alkanes (analytical grade ≥98% purity) were purchased from EAD Milliore Corporation, Burlington, MA, USA. A mixture of the alkanes from C_6_–C_32_ was prepared for the identification of the different alkanes. Formic acid (≥98%), acetic acid (≥99.8%), propanoic acid (≥99.5%), butanoic acid (≥99%), nonanoic acid (≥97%), decanoic acid (≥98%), hexadecanoic acid (≥99%), octadecanoic acid (≥98.5%), squalene (≥98%), cholesterol (≥99%), styrene (≥99%), acetaldehyde (≥99.5%), hexanal (≥97%), heptanal (≥95%), *p*-cymene (≥97%), and caryophyllene (≥97%) were purchased from Sigma-Aldrich (St. Louis, MO, USA).

### 3.2. Insects

Ant colonies were collected from mango trees at the Muhaka field station (−4°32′41′′ S, 39°52′44′′ E) of the International Centre of Insect Physiology and Ecology (*icipe*) in Kwale County, Kenya. Tree branches with nests were cut and placed in plastic containers (45 × 30 × 15 cm^3^), with fine netting attached to the lid for ventilation. The nests were transferred to potted mango seedlings in a greenhouse at *icipe*’s main campus (−1°22′17′′ S, 36°89′65′′ E) in Nairobi, Kenya. Pots were thereafter placed in the center of a tray filled with soapy water to confine the ants to the host plant as well as to keep predatory insects from getting access to the ants. The plants were watered and branches pruned regularly to prevent the ants from escaping. The plants were maintained under natural lighting in the greenhouse (12 light:12 dark) at 29 ± 2 °C and 65 ± 5 RH%, and the ants were fed on a 10% sugar solution and freshly killed fruit flies (adult *Bactrocera dorsalis* and *Ceratitis cosyra*) twice a week. A total of seven colonies (four for solvent extraction and three for headspace sampling) on different seedlings were maintained and used for GC-MS analysis.

### 3.3. Extraction of Ant Gland Contents

**Figure 1:**
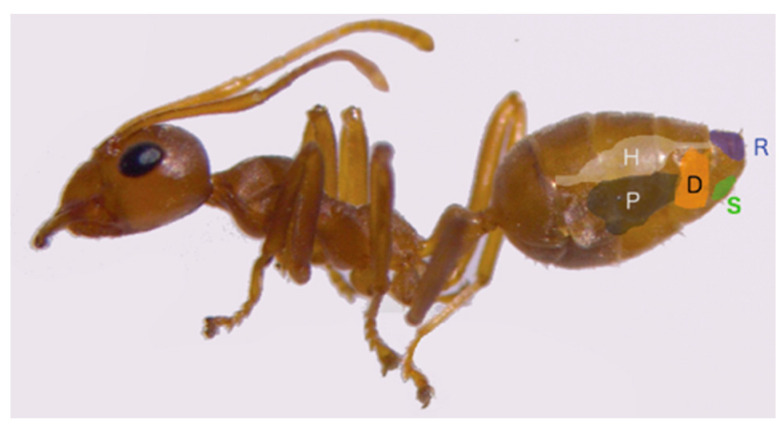
Schematic diagram of worker African weaver ant’s exocrine glands in the abdomen; (P) poison, (D) Dufour’s, (S) sternal, (R) rectal, and (H) hindgut.

To extract glandular contents, adult major worker ants were immobilized on ice and abdomens removed using dissection scissors. The four different abdominal glands (10 Dufour’s glands and 30 sternal, rectal, and poison glands (Figure 1)) were extracted by carefully removing all the cuticles (dorsal and sternal) and the remnant tissues. To extract the rectal gland, the whole of the hindgut was removed, and for the sternal gland, the last sternite was included to ensure the removal of all the contents. To avoid contamination among gland components, the water in the petri dish was replaced after each dissection and the forceps cleaned upon removal of each gland, using cotton soaked in distilled water followed by washing with acetone. The dissections were carried out in a petri dish containing distilled water under a simple microscope with a magnification power ranging from 10× to 30× using fine forceps (5SF, 11250-00 Inox-Biology CE). The glands were placed in vials containing 2000 µL of hexane and vortexed for 10 s, followed by 30 min of sonication (ultrasonic bath at room temperature) to agitate particles in the sample. After this, the suspension was centrifuged at 15,000 rpm for 30 min at 4 °C and the supernatant was filtered through glass wool. Sodium sulfate (NaSO_4_) was added to remove water in the samples, vortexed, and centrifuged at 15,000 rpm for 10 min at 4 °C. The solutions were evaporated to dryness and reconstituted in 100 µL of hexane.

Each extraction was repeated four times using different glands obtained from different ant populations for each extraction, and samples were stored at −80 °C until use.

### 3.4. Headspace Sampling by SPME

Collection of headspace volatiles was performed using a manual solid-phase microextraction (SPME) fiber, with a layer of polydimethylsiloxane (PDMS) obtained from Supelco Co (Bellefonte, PA, USA; Taufkirchen, Germany). Volatile contents of Dufour’s and poison glands were collected from 10 glands each, combined and obtained from different ant populations. The gland reservoir was punctured and introduced into 2 mL vials (Supelco) sealed with a Teflon/silicon septum (Supelco) cap containing an insert. Each fiber was conditioned at 250 °C for 15 min before use by putting it into the injector port of a GC instrument operated in split mode with septum purge and purge flow set at 3 mL/min. Volatiles were collected from the glands by piercing the vial cap with the sample using a needle. The fiber was then exposed to the headspace, 2 mm above the sample, for 1 h. The fiber was drawn into the protecting needle before retracting the SPME fiber holder, and volatile collection was repeated three times using a different fiber. For all samples, a blank collection was made using the 2 mL vial and repeated three times.

### 3.5. Coupled Gas Chromatography–Mass Spectrometry (GC-MS) Analysis

Compounds in the glands were identified by coupled gas chromatography–mass spectrometry (GC-MS) on an HP 7890A series gas chromatograph (Agilent Technologies, Wilmington, NC, USA) linked to an HP 5975C mass spectrometer (Agilent Technologies, Wilmington, NC, USA) operated in electron ionization mode (70 eV). The instrument was equipped with a non-polar HP-5MS capillary column (30 m × 0.25 mm i.d.; 0.25 μm film thickness; J &W Scientific, Folsom, CA, USA). Helium was used as the carrier gas at 1.2 mL min^−1^. One microliter of each sample was injected in splitless mode at 35 °C for 5 min, increasing the temperature to 280 °C at 10 °C min^−1^. The solvent hexane used for extraction was analyzed similarly. The injector and the detector were held isothermal at 280 °C for 35 min. The ion source temperature was 230 °C. Electron ionization mass spectra were acquired at 70 eV within a mass range of 38–550 Daltons (Da) during a scan time of 0.73 scans s^−1^. Volatile compounds were identified using their retention times and mass fragmentation spectra against authentic standards (those available). Others were tentatively identified using matches of three mass spectral libraries: Adams, Chemoecol, and National Institute of Standards and Technology (NIST) (MSD Chemstation E.02.00.493, MS HP, USA). A blend of alkanes (C_6_–C_32_) was injected to calculate the retention index (RI) = [TR(X) − TR(*n*)]/[TR(*n* + 1) − TR(*n*)]*100 + (100*n), where TR(X) is the retention time of the studied product, TR(*n*) is the retention time of the alkane with *n* carbons that eluted before X, and TR(*n* + 1) is the retention time of the alkane of *n* + 1 carbons that eluted after X. Components identified in the blank volatile collections were excluded from the analysis. Serial dilutions of the authentic standard undecane (0.1–100 ng/µL) were analyzed by GC-MS to generate a linear calibration curve (peak area vs. concentration), which gave the coefficient of determination (R^2^ = 0.9937). This regression equation was used for the external quantification of the different volatile organic compounds (VOCs).

### 3.6. Statistical Analysis

The relative amounts of compounds were computed by dividing the peak area of the compound by the sum of the peak areas of all compounds. The data were presented as the average percentage of replicates. Relative standard deviations (RSDs) were determined from (1) four different colonies for Table 1 and (2) three colonies for Table 2, using the following formula RSD = (S*100)/*X*, where *S* is the standard deviation and *X* the mean of peak areas of compounds.

## 4. Conclusions

Our results demonstrate that Dufour’s and poison glands of *O. longinoda* secrete a wider range of compounds than previously reported. Moreover, we found some compounds specific to the secretions of these two glands plus the sternal and rectal glands. As such, our results suggest that using a combination of analytical chemistry techniques, SPME and solvent extraction, could be a more effective method to unravel the composition of ant gland secretions. Our findings of the compositions of the gland secretions are similar and different in some cases between *O. longinoda* and other ant species, which may be important for chemotaxonomic studies and phylogenetic relationships. Additionally, future research should investigate the roles of these additional components identified in the gland secretions in the biology and physiology of *O. longinoda*.

## Figures and Tables

**Table 1:** Percentage compositions and amounts of compounds detected in Dufour’s, poison, sternal, and rectal gland extracts of major workers of *Oecophylla longinoda*.

	Content (%) Mean ± SE						Absolute Amount (ng)			
Compounds	RT	RI	Dufour’s	Poison	Rectal	Sternal	Dufour’s	Poison	Rectal	Sternal
***Hydrocarbons***										
**Heptane** ^a^	3.82	700	-	-	-	1.65 ± 0.32	-	-	-	3.4
**Octane** ^a^	6.51	800	-	0.08 ± 0.06	0.06 ± 0.15	0.09 ± 0.01	1.79	0.91	0.84	0.86
**Unidentified branched alkane 1** ^b^	7.85	842	-	-	-	0.19 ± 0.10	-	-	-	1.04
**Nonane** ^a^	9.53	900	0.14 ± 0.02	1.14 ± 0.47	0.65 ± 0.21	-	0.94	3.42	1.89	
Decane ^a^	11.88	1000	0.90 ± 0.08	-	0.37 ± 0.89	0.23 ± 0.10	2.16	-	1.39	1.09
**Unidentified alkene 1** ^b^	12.25	1041	0.16 ± 0.08	-	-	-	1.01	-	-	-
Undecane ^a^	13.38	1100	39.64 ± 1.06	-	8.62 ± 3.27	7.33 ± 6.46	68.64	-	16.18	12.6
Dodecane ^a^	14.84	1200	0.94 ± 0.05	0.28 ± 0.14	1.31 ± 1.15	0.80 ± 0.35	2.27	1.37	3.07	2.01
**Unidentified branched alkane 2** ^b^	16.07	1276	-	-	-	0.74 ± 0.27	-	-	-	1.92
**Unidentified branched alkane 3** ^b^	16.25	1288	-	-	-	0.85 ± 0.35	-	-	-	2.1
**Unidentified alkene 2** ^b^	16.29	1291	0.51 ± 0.07	-	-	-	1.54	-	-	-
**Unidentified alkene 3** ^b^	16.31	1292	1.61 ± 0.13	-	-	-	3.38	-	-	-
Tridecane ^a^	16.61	1300	4.72 ± 0.24	0.77 ± 0.12	4.65 ± 3.72	3.12 ± 0.60	8.59	2.53	9.05	5.78
Tetradecane ^a^	17.48	1400	0.37 ± 0.02	1.99 ± 0.85	1.35 ± 0.45	0.25 ± 0.03	1.3	5.42	3.15	1.13
Pentadecane ^a^	18.69	1500	7.07 ± 0.09	1.66 ± 0.15	1.34 ± 0.59	4.18 ± 1.28	12.54	4.63	3.13	7.49
**Unidentified alkene 4** ^b^	18.99	1520	0.14 ± 0.02	-	-	-	0.96	-	-	-
Hexadecane ^a^	20.48	1600	0.07 ± 0.01	1.13 ± 0.19	1.37 ± 0.32	0.50 ± 0.12	0.84	3.39	3.19	1.54
**Unidentified alkene 5** ^b^	21.05	1663	1.07 ± 0.12	-	-	-	2.48	-	-	-
**Unidentified alkene 6** ^b^	21.12	1669	0.86 ± 0.02	-	-	-	2.13	-	-	-
Heptadecane ^a^	21.47	1700	0.11 ± 0.01	-	3.70 ± 1.03	3.44 ± 1.79	0.89	-	7.36	6.29
Octadecane ^a^	22.46	1800	0.09 ± 0.02	3.84 ± 1.41	1.08 ± 0.32	0.56 ± 0.09	0.88	3.08	2.66	1.63
Nonadecane ^a^	23.44	1900	1.16 ± 0.11	3.27 ± 0.86	1.81 ± 0.66	2.49 ± 0.37	2.74	8.45	3.97	4.75
**Unidentified alkene 7** ^b^	23.82	1949	0.24 ± 0.03	-	-	-	1.14	-	-	-
Eicosane ^a^	24.31	2000	0.55 ± 0.06	4.84 ± 0.99	2.43 ± 1.03	1.77 ± 0.35	1.67	12.16	5.08	3.59
**Unidentified alkene 8** ^b^	24.95	2070	1.19 ± 0.07	-	-	-	2.67	-	-	-
Heneicosane ^a^	25.01	2100	12.47 ± 0.42	5.15 ± 1.61	8.38 ± 1.75	16.5 ± 2.42	22.14	12.89	15.75	27.58
Docosane ^a^	25.97	2200	1.77 ± 0.11	3.71 ± 0.72	3.66 ± 0.40	2.32 ± 0.15	3.73	9.49	7.29	4.48
**Unidentified alkene 9** ^b^	26.8	2239	2.57 ± 0.06	-	4.79 ± 2.38	-	4.82	-	9.31	-
Tricosane ^a^	26.98	2300	16.84 ± 0.33	8.77 ± 2.18	12.78 ± 3.38	24.2 ± 2.17	28.99	21.45	23.64	40.07
Unidentified alkene 10 ^b^	27.53	2364	0.35 ± 0.03	5.13 ± 1.45	4.07 ± 0.73	3.78 ± 0.53	1.33	12.89	8.02	6.85
**Tetracosane** ^a^	27.61	2400	0.25 ± 0.03	-	-	-	1.11	-	-	
Pentacosane ^a^	28.61	2500	1.10 ± 2.39	4.16 ± 1.78	4.58 ± 0.81	3.91 ± 0.54	2.54	10.56	8.94	7.06
**Hexacosane** ^a^	29.87	2600	-	6.48 ± 1.16	3.04 ± 2.07	3.65 ± 0.47	-	16.05	6.16	6.63
**Heptacosane** ^a^	30.43	2700	-	7.06 ± 1.99	4.95 ± 0.29	2.34 ± 0.35	-	17.42	9.6	4.52
**Octacosane** ^a^	30.79	2800	-	4.80 ± 0.46	2.68 ± 0.61	3.56 ± 0.35	-	12.08	5.52	6.49
**Nonacosane** ^a^	33.39	2900	-	4.83 ± 0.88	8.06 ± 1.82	5.30 ± 1.18	-	10.6	15.16	9.31
**Squalene** ^a^	33.57	2934	-	4.18 ± 0.28	3.66 ± 0.53	2.42 ± 0.43	-	12.15	7.28	4.65
**Triacontane** ^a^	34.1	3000	-	4.02 ± 1.25	3.91 ± 0.43	2.57 ± 0.47	-	10.23	7.73	4.89
**Hentriacontane** ^a^	34.89	3100	-	6.20 ± 2.54	4.74 ± 1.97	-	-	15.38	9.22	-
***Carboxylic acids***										
**Decanoic acid** ^a^	16.45	1301	0.77 ± 0.14	-	-	-	1.74	-	-	-
**Hexadecanoic acid** ^a^	23.68	1933	0.40 ± 0.05	-	-	-	1.13	-	-	-
**Unidentified carboxylic acid 1** ^b^	25.75	2156	1.66 ± 0.19	-	-	-	2.43	-	-	-
**Octadecanoic acid** ^a^	25.94	2177	0.30 ± 0.03	-	-	-	1.25	-	-	-
***Terpene***										
**α-Cedrene** ^a^	18.11	1422	-	-	-	0.96 ± 0.46		-	-	-
***Alcohol***										
**Unidentified alcohol 1** ^b^	24.81	2055	-	3.84 ± 1.41	-	-		9.8	-	-
***Sterols***										
**Cholesterol** ^a^	35.56	3074	-	6.10 ± 2.09	-	-		15.16	-	-
**Unidentified sterol 1** ^b^	36.14	3094	-	4.07 ± 1.95	-	-		10.33	-	-
**Campesterol** ^a^	37.6		-	5.34 ± 1.00	-	-		13.34	-	-

RT (min): retention time in minutes; SE: standard error; ^a^ compounds identified by the injection of synthetic standards; ^b^ compounds tentatively identified by gas chromatography–mass spectrometry (GC-MS) library data only. RI: retention index relative to C_6_−C_32_ n-alkanes on an HP-5 MS column. Compound names in bold are additional components reported for the first time in gland secretions. RSD < 10, green; RSD < 50, yellow; RSD < 100, red; and RSD > 100, no color.

**Table 2:** Percentage compositions and amounts of headspace volatiles of Dufour’s and poison glands of major workers of *O. longinoda.*

Content (%) Mean ± SE					Absolute Amount (ng)	
Compounds	RT	RI	Dufour’s	Poison	Dufour’s	Poison
***Hydrocarbons***						
Nonane ^a^	9.53	900	0.19 ± 0.12	0.02 ± 0.01	7.6	0.82
Decane ^a^	11.88	1000	3.21 ± 0.85	-	117.19	-
**Unidentified alkene 1** ^b^	12.83	1077	0.13 ± 0.08	0.35 ± 0.24	5.47	2.46
Undecane ^a^	13.38	1100	59.29 ± 4.40	32.52 ± 8.99	2149.44	164.64
**Unidentified alkene 2** ^b^	14.44	1167	0.14 ± 0.06	0.16 ± 0.10	5.89	1.51
Dodecane ^a^	14.84	1200	3.22 ± 0.48	0.82 ± 0.20	117.52	4.85
**Unidentified alkene 3** ^b^	14.56	1245	0.47 ± 0.21	-	17.63	-
**Unidentified alkene 4** ^b^	15.7	1251	0.15 ± 0.11	-	6.02	-
**Unidentified alkene 5** ^b^	15.83	1260	0.85 ± 0.39	0.33 ± 0.08	31.67	2.37
**Unidentified alkene 6** ^b^	15.94	1265	3.89 ± 0.80	1.13 ± 0.36	141.75	6.39
Tridecane ^a^	16.61	1300	11.29 ± 1.50	4.30 ± 1.37	109.73	22.4
**Unidentified alkene 7** ^b^	17.2	1352	0.25 ± 0.14	-	9.92	-
Tetradecane ^a^	17.48	1400	1.14 ± 0.17	0.39 ± 0.23	42.21	2.68
Pentadecane ^a^	18.69	1500	8.47 ± 3.20	6.08 ± 1.60	307.56	31.35
**Unidentified alkene 8** ^b^	19.62	1538	0.12 ± 0.05	-	4.97	-
Hexadecane ^a^	19.84	1600	0.12 ± 0.06	0.20 ± 0.19	5.01	1.72
**Unidentified alkene 9** ^b^	21.12	1669	0.52 ± 0.06	0.37 ± 0.06	19.51	2.57
Heptadecane ^a^	21.47	1700	0.12 ± 0.06	-	5.13	-
Octadecane ^a^	22.46	1800	0.11 ± 0.08	0.12 ± 0.03	4.85	1.34
Eicosane ^a^	24.31	2000	0.23 ± 0.11	-	9.02	-
Heneicosane ^a^	25.01	2100	0.18 ± 0.11	0.08 ± 0.05	7.16	1.12
Docosane ^a^	25.97	2200	0.36 ± 0.29	0.24 ± 0.23	13.75	1.93
Tricosane ^a^	26.98	2300	0.13 ± 0.10	0.25 ± 0.21	5.49	1.95
Tetracosane ^a^	27.61	2400	0.46 ± 0.32	0.35 ± 0.23	17.25	2.47
***Carboxylic acids***						
Formic acid ^a^	1.9		-	38. 47 ± 8.85	-	194.59
**Acetic acid** ^a^	2.37		0.68 ± 0.47	3.45 ± 0.32	25.39	18.11
**Propanoic acid** ^a^	3.42		0.16 ± 0.10	0.43 ± 0.08	6.61	2.9
**2-Propenoic acid** ^a^	3.63		0.01 ± 0.01	-	6.61	-
**2-Methylpropanoic acid**	5.22		0.11 ± 0.07	0.03 ± 0.02	1.1	0.88
**Butanoic acid** ^a^	6.05		0.17 ± 0.15	0.10 ± 0.07	4.62	1.22
**3-Methylbutanoic acid** ^a^	7.81		0.06 ± 0.05	0.09 ± 0.06	6.93	1.19
**2-Methylhexanoic acid** ^a^	8.04		-	0.17 ± 0.07	-	1.58
**Unidentified carboxylic acid 1** ^b^	17.72		-	0.52 ± 0.27	-	3.31
***Aldehydes***						
**Acetaldehyde** ^a^	1.13		0.21 ± 0.16	2.68 ± 1.09	8.21	14.2
**2-Methylbutanal** ^a^	2.84		-	0.08 ± 0.05	-	1.14
**Hexanal** ^a^	6.34		0.03 ± 0.02	0.14 ± 0.05	1.91	1.44
**Heptanal** ^a^	9.09		-	0.06 ± 0.01	-	1.04
**Unidentified aldehyde** 1 ^b^	21.28		0.09 ± 0.06	-	4.12	
***Terpenes***						
***p*-Cymene** ^a^	11.6	1000	-	0.07 ± 0.04	-	1.06
**Caryophyllene** ^a^	17.8	1400	0.13 ± 0.11	-	5.28	-
**Unidentified sesquiterpene 1** ^b^	19.08	1500	-	0.18 ± 0.11	-	1.64
***Alcohols***						
**Unidentified alcohol 1** ^b^	17.87	1400	-	0.10 ± 0.17	-	2.24
**1-Tridecanol** ^b^	18.43	1444	0.72 ± 0.16	-	27.31	-
***Ketones***						
**Acetone** ^a^	1.61		0.01 ± 0.00	-	0.89	-
**Unidentified ketone 1** ^b^	13.48			0.37 ± 0.26	-	2.1
***Benzenoid***						
**Styrene** ^a^	8.78	878	-	0.12 ± 0.12	-	1.3
***Miscellaneous***						
Unknown 1	1.52		-	3.03 ± 2.06	-	-
Unknown 2	5.19		-	0.11 ± 0.10	-	-
Unknown 3	11.04		-	0.25 ± 0.06	-	-
Unknown 4	15.65		-	0.16 ± 0.13	-	-
Unknown 5	17.96		-	0.40 ± 0.15	-	-
Unknown 6	18.07		-	0.25 ± 0.13	-	-
Unknown 7	18.18		-	0.26 ± 0.25	-	-
Unknown 8	18.25		-	0.49 ± 0.29	-	-
Unknown 9	19.21		-	0.33 ± 0.23	-	-

RT (min): retention time in minutes; SE: standard error; ^a^ compounds identified by the injection of synthetic standards; ^b^ compounds tentatively identified by GC-MS library data only. RI: Retention index relative to C_6_−C_32_ n-alkanes on an HP-5 MS column. Compound names in bold are additional components reported for the first time in gland secretions. RSD < 10, green; RSD < 50, yellow; RSD < 100, red; and RSD > 100, no color.

## Data Availability

The data presented in this study are openly available in [FigShare] at [doi.org/10.6084/m9.figshare. 13711726.v1].

## Sample Availability

Samples are not available from the authors.

## References

[1] Morgan E.D. (2008). Chemical sorcery for sociality: Exocrine secretions of ants (Hymenoptera: Formicidae). Myrmecol News.

[2] Sherman G., Visscher P.K. (2002). Honeybee colonies achieve fitness through dancing. Nature.

[3] Regnier F.E., Nieh M., Holldobler B. (1973). The volatile Dufour’s gland components of the harvester ants, *Pogonomyrmex rugosus* and *P. barbatus*. J. Insect. Physiol..

[4] Tappey H.J., Voegtle H.L., Miras H.M., Weatherford R.G., Spande T.F., Garraffo H.M., Snelling R.R. (2007). Venom chemistry of the ant, *Myrmicaria melanogaster* from Brunei. J. Nat. Prod..

[5] Vander Meer R.K., Preston C.A., Choi M.-Y. (2010). Isolation of a Pyrazine alarm pheromone component from the fire Ant, *Solenopsis invicta*. J. Chem. Ecol..

[6] Lucas C., Pho D.B., Jallon J.M., Fresneau D. (2005). Role of cuticular hydrocarbons in the chemical recognition between ant species in the *Pachycondyla villosa* species complex. J. Insect Physiol..

[7] Walter F., Fletcher D.J.C., Chautems D., Cherix D., Keller L., Francke W., Fortelius W., Rosengren R., Vargo E.L. (1993). Identification of the sex pheromone of an ant, *Formica lugubris* (Hymenoptera, Formicidae). Naturwissenschaften.

[8] Attygalle A.B., Mutti A., Rohe W., Maschwitz U., Garbe W., Bestmann‚ H.J. (1998). Trail pheromone from the Pavan gland of the ant, *Dolichoderus thoracicus* (Smith). Naturwissenschaften..

[9] Janssen E., Hölldobler B., Bestmann H.J. (1999). A trail pheromone component of the African stink ant, *Pachycondyla* (Paltothyreus) *tarsata* Fabricius (Hymenoptera: Formicidae: Ponerinae). Chemoecology.

[10] Kern F., Klein R.W., Janssen E., Bestmann H.J., Attygalle A.B., Schäfer D., Maschwitz U. (1997). Mellein, a trail pheromone component of the ant *Lasius fuliginosus*. J. Chem. Ecol..

[11] Crozier R.H., Newey P.S., Schlüns E.A., Robson S.K.A. (2010). A masterpiece of evolution: *Oecophylla* weaver ants (Hymenoptera: Formicidae). Myrmecol. News..

[12] Hölldobler B., Wilson E.O. (1978). Multiple recruitment systems of African weaver ant *Oecophylla longinoda* (Latreille) (Hymenoptera Formicidae). Behav. Ecol. Sociobiol..

[13] Bradshaw J.W.S., Baker R., Howse P.E. (1979). Chemical composition of the poison apparatus secretions of the African weaver ant, *Oecophylla longinoda*, and their role in behaviour. Physiol. Entomol..

[14] Kempraj V., Park S.J., DE Faveri S., Taylor P.W. (2020). Overlooked Scents: Chemical profile of soma, volatile emissions and trails of the green tree ant, *Oecophylla smaragdina*. Molecules.

[15] Keegans S.J., Billen J., Morgan E.D. (1991). Volatile secretions of the green tree ant, *Oecophylla smaragdina* (Hymenoptera: Formicidae). Comp. Biochem. Physiol. B..

[16] Witte V., Abrell L., Attygalle A.B., Wu X., Meinwald J. (2007). Structure and function of Dufour gland pheromones from the crazy ant, *Paratrechina longicornis*. Chemoecology.

[17] Attygalle A.B., Vostrowsky O., Bestmann H.J., Morgan E.D. (1987). New chemicals from the Dufour gland of the formicine ant *Lasius niger* (Hymenoptera: Formicidae). Insect Biochem..

[18] Jackson B.D., Billen J.P.J., Morgan E.D. (1989). Dufour gland contents of three species of *Myrmecia* (Hymenoptera: Formicidae), primitive ants of Australia. J. Chem. Ecol..

[19] Gokcen O.A., Morgan E.D., Dani F., Agosti D., Wehner R. (2002). Dufour gland contents of ants of the *Cataglyphis bicolor* group. J. Chem. Ecol..

[20] Bergstrom G., Lofqvist J. (1968). Odour similarities between the slave-keeping ants, *Formica sanguinea* and *Polyergus rufescens* and their slaves *Formica fusca* and *Formica rufibarbis*. J. Insect Physiol..

[21] Falótico T., Labruna M.B., Verderane M.P., De resende Briseida D., Izar P., Ottoni E.B. (2007). Repellent Efficacy of Formic Acid and the Abdominal Secretion of Carpenter Ants (Hymenoptera: Formicidae) Against Amblyomma Ticks (Acari: Ixodidae). J. Med. Entomol..

[22] Frederickson M.E., Greene M.J., Gordon D.M. (2005). ‘Devil’s gardens’ bedevilled by ants. Nature.

[23] Löfqvist J. (1976). Formic acid and saturated hydrocarbons as alarm pheromones for the ant, *Formica rufa*. J. Insect. Physiol..

[24] Vick K., Drew W.A., McGurk D.J., Eisenbraun E.J., Waller G.R. (1969). Identification of hydrocarbons from *Novomessor cockerelli*. Ann. Entomol. Soc. Am..

[25] Dani F.R., Jones G.R., Morgan E.D., Turillazzi S. (2003). Reevaluation of the chemical secretion of the sternal glands of Polistes social wasps. Ethol. Ecol. Evol..

